# Supplementing factual information with patient narratives in the cancer screening context: a qualitative study of acceptability and preferences

**DOI:** 10.1111/hex.12357

**Published:** 2015-03-01

**Authors:** Kirsty F. Bennett, Christian von Wagner, Kathryn A. Robb

**Affiliations:** ^1^ Division of Psychiatry University College London London UK; ^2^ Department of Epidemiology and Public Health University College London London UK; ^3^ Institute of Health and Wellbeing University of Glasgow Glasgow UK

**Keywords:** bowel cancer, cancer screening, narratives, patient information, qualitative study

## Abstract

**Objective:**

To explore people's responses to narrative information in the context of colorectal cancer screening.

**Design:**

Nineteen in‐depth interviews were conducted with men and women (aged 45–59). Participants were given two types of colorectal screening information to read: factual and narrative. Participants gave their views on both types of information. Data were analysed using Framework Analysis.

**Results:**

The most frequent responses to the narrative information were that they were reassuring, made colorectal screening more vivid, participants could relate to the people in the stories and they liked the range of narratives presented. Despite the narrative information being seen as more persuasive by some, this was not regarded as manipulative or negative. Both types of information were seen as equally credible. Participants felt a combination of facts and narratives would be useful when considering an offer of colorectal cancer screening.

**Conclusion:**

Overall, participants were positive about the addition of narrative information to the currently provided factual information about colorectal cancer screening. Supplementing existing factual information with narrative information may provide participants with a more complete understanding of participation in colorectal cancer screening when considering an offer to be screened.

## Introduction

Patient narratives or stories are increasingly used to provide health information to patients and the public.^1^, ^2^ The content and form of patient narrative messages varies enormously,^3^ but it can be usefully defined as, ‘…concrete, emotionally interesting information such as a first person account of someone who came to experience a particular condition that may also affect the message recipient’.^4^ Prominent examples of the use of patient narratives include the Centers for Disease Control and Prevention's ‘Tips from former smokers’ campaign,^5^ and the Witness Project which presented cancer survivors talking about their experiences to promote early detection of breast cancer among African American women.^6^

Traditionally, health information has taken a more didactic approach with the presentation of facts and statistics.^1^ The NHS Cancer Screening Programmes encourage individuals to make an informed choice about whether to participate in screening, by providing balanced information about the risks and benefits.^7^ This approach assumes that people make decisions by rationally weighing up the pros and cons of a behaviour but in reality we know that decisions are frequently also informed by experiential and affective information.^8^, ^9^, ^10^ Indeed, people may have a preference for either rational/deliberative thinking or more experiential thinking.^10^ Supplementing factual information, which is essential in making health decisions, with experiential or narrative information may not only engage a larger number of people in the information, but may also provide important emotional or social information which is typically lacking in routine health information resources. It has also been suggested that narrative information is more easily processed^10^ and may be particularly beneficial to people with low literacy skills.^11^, ^12^

Research on the use of patient narratives in health‐care decision‐making is still in its early stages, and it is not yet clear whether personal stories can increase the effectiveness of decision aids.^13^ To date, most work has taken a quantitative approach to assess the impact of narrative information – usually in relation to more factual information – on cognitions, intentions and behaviour.^4^, ^14^, ^15^, ^16^, ^17^ However, the use of patient narratives as a source of information in decision‐making has been regarded as controversial as they can appear more powerful and persuasive than factual presentations of information, and selecting stories that provide an appropriate ‘balance’ of experiences remains a challenge.^2^, ^18^, ^19^

In addressing this controversy, little research has considered people's preferences for and acceptance of narrative information. In a survey of a mostly female (92%), online weight loss community there was interest in sharing and receiving experiences of colorectal cancer screening.^20^ The present study sought to explore people's preferences and perceived acceptability of narrative information in more depth by taking a qualitative approach.

The context of the study was colorectal cancer screening. The English colorectal cancer screening programme using the home‐based Faecal Occult Blood test (FOBt) was introduced in 2006 and is initiated by a written invitation letter and a factual information leaflet, ‘Bowel Cancer Screening: the facts’^21^ – the term ‘bowel’ is more commonly used than colorectal in the UK. This study examined people's responses to narrative information about colorectal screening as a supplement to an adapted version of the currently provided factual information. Although narratives can be used as a decision aid, the purpose of the current study was to consider their use in the context of engaging more people in bowel screening rather than aiding informed decision‐making.

## Methods

### Participants and procedures

Participants (*n* = 19) were purposively sampled to represent men and women within the target age range from a population‐based survey on colorectal cancer screening in which participants had responded that they were willing to be contacted about future research. Participants were aged 45–59, to avoid contacting individuals who may have experienced the FOBt (it begins at age 60 in England), but who were approaching the screening age. Ethical approval was obtained from the NHS West London REC 2 Research Ethics Proportionate Review Sub Committee.

All interviews took place at University College London between March and May 2011. Informed consent was obtained before the start of each interview. Participants were asked to read a short piece of information on the FOBt, which included the purpose of the test, how the test worked, what would happen if an abnormal result was found and a picture of the screening kit. Following this, participants were presented with the factual and narrative information and asked to choose which information they would like to read first. After participants had read both the factual and narrative information, they were interviewed in‐depth using a topic guide. The topic guide addressed participants’ thoughts and feelings in response to the factual and narrative information. Participants were asked to compare the two materials and to consider which type of information they would like if they were considering a screening offer. Finally, we asked participants what they thought could be done to improve the information.

### Information materials

The factual information was adapted from the NHS Bowel Cancer Screening Programme leaflet.^19^ It covered why colorectal cancer screening is important, what the NHS Bowel Cancer Screening Programme is, what the colon does, what colorectal cancer is, who is at risk of developing colorectal cancer, how the screening test works, how the screening is carried out, possible results and what they mean, and some information on colonoscopy follow‐up if the test is positive. The factual information was presented on two A4 pages and was 734 words in length. This was considerably shorter than the NHS Bowel Cancer Screening Programme leaflet which is presented across 15 pages in an A5 booklet. The Flesch Reading Ease score was 62.6 (scores between 60 and 70 are considered acceptable, higher scores indicate a more easily understood document). We chose to abridge the factual information and present it on two A4 pages to reduce the burden on our participants. We reduced the information on having a colonoscopy and did not include information on the symptoms or treatment of bowel cancer because we felt these were less relevant in the context of this study.

Narratives can be presented in many different ways^3^ and be framed to emphasize the benefits of adopting a behaviour or the costs of failing to adopt a behaviour.^22^ A narrative or ‘testimonial’ recommending adopting a behaviour is likely to have a different impact and potentially be more persuasive on decision‐making than a narrative describing an individual's thoughts and experience. For that reason, the narrative information was selected from the publicly available information resource (http://www.healthtalk.org/) which provides, ‘free, reliable information about health issues, by sharing people's real‐life experiences.’

The narrative information for this study consisted of four people's experiences of doing the FOBt. Many more experiences of FOBt were available from the healthtalk.org website but again we wished to reduce the burden on our participants. Two men and two women were selected to represent a range of views and outcomes. As the rationale for the study was to examine ways to potentially increase uptake of bowel screening rather than informed decision‐making, only narratives from people who had completed the test were included. Two people had normal FOBt results, one was diagnosed with colorectal cancer having done the FOBt and follow‐up colonoscopy, and one person initially had a normal FOBt result but a subsequent test was abnormal and he had a colonoscopy where non‐malignant polyps were removed. One participant described feeling apprehensive ‘didn't quite like the idea of it’, and another initially declined the invitation because she thought it was disgusting but did complete it the third occasion she was invited (4 years after the initial invitation).

The narrative information covered each individual's views, thoughts and feelings of the FOBt and their experience of doing the test. Each narrative also included a small photograph of the person, their ethnic background, profession, marital status and whether they had children. It was felt including this information and a photo would make the narratives more engaging. Additionally, Social Cognitive Theory suggests that seeing people similar to oneself can strengthen self‐efficacy of carrying out a behaviour.^23^ The narrative information was also two A4 pages in length, but with 1077 words contained more words than the factual information. The Flesch Reading Ease test score was 67.1, indicating that the narrative information was slightly easier to read than the factual information. Copies of both the factual and narrative information are available from the corresponding author.

### Analysis

Interviews were digitally recorded, transcribed verbatim and checked twice for accuracy. Data were analysed using framework analysis, ‘…a matrix based analytic method which facilitates rigorous and transparent data management’.^24^ Framework analysis allows organization of data according to key themes and concepts and each thematic framework comprises of main themes and subthemes. After familiarization with the transcripts, recurrent themes were identified and applied to the data. Data were then extracted from the transcripts and charted into themes, each displayed in a separate table, where each participant was allocated a row, with columns representing subthemes. The thematic framework was an independent and iterative process until KB and KR were satisfied that the framework was appropriate for the data.

## Results

Demographic details of the participants are summarized in Table 1. The average age was 49.3 years (range 45–55 years). Slightly more women (*n* = 11) than men (*n* = 8) participated. The sample was predominantly White British (*n* = 14) with four participants describing themselves as White ‘other’, and one participant who was of mixed ethnicity. Two participants had no qualifications, seven had some school‐level or vocational qualifications, and ten had a university degree. One participant had had cancer and seventeen participants had family or friends who had had cancer.

**Table 1:** Demographic characteristics of the sample. Numbers are ‘*n*’ unless otherwise stated

Demographic characteristics	
Age mean years (range) – 1 age missing	49.3 (45–55)
Gender	
Male	8
Female	11
Ethnicity	
White British	14
White – Not British or Irish	4
Mixed	1
Education	
No formal qualifications	2
School‐level or vocational qualifications	7
University qualification	10
Experience of cancer among family and friends	
Yes	17
No	0
Not sure	2
Personal experience of cancer	
Yes	1
No	18

### Responses to the factual information

Fewer comments were made about the factual than the narrative information. Responses to the factual information were generally positive. A common theme was how well written, clear and easy to understand the information was, with relevant scientific terms explained: ‘I think this one [factual] is particularly good because I've read a lot of things like this and I'm struck by how good and clear it is’ (F11, 48y – participant ID numbers use M and F to denote male and female, respectively); ‘…it's very straightforward; it's very good, very easy to understand…I thought it was great.’ (F6, 47y).

Participants felt the factual information was educational and provided general information about colorectal screening which people may not know. Participants were ‘shocked’ and ‘surprised’ by the high incidence of colorectal cancer and seemed unaware that it is a common cancer: ‘….I didn't realize that so many people would suffer from it.’ (M5, 45y).

Five participants made negative comments about the factual information which included that it might make people anxious or worried (*n* = 3) that statistics can be harder to understand: ‘it's facts and figures…you kind of get a bit, not overwhelmed, but you know it doesn't always sink in so much.’ (F4, 47y) and that they seemed too academic with too many technical terms, ‘I thought sometimes it was a bit too much, I didn't need the references, where the information came from, that was a bit academic. I mean, “a polyp is sometimes known as an adenoma”,… I don't think I necessary needed to know.’ (M1, 53y).

### Responses to the narrative information

Responses to the narrative information were also generally positive and a number of themes emerged.

#### Identification with people in the stories

A common theme among participants (9/19) was that they could relate to and identify with the people in the narratives particularly because the stories included background information which helped to illustrate that they were ‘ordinary’ people: ‘…saying what they do and that they're married and they have children that makes you identify a little bit more with them.’ (M1, 53y); ‘..[it's] good to hear everyday people's feedback and what their kind of feelings were about it…. because that's obviously what you kind of relate to if you had to do it yourself’ (F4, 47y). It was felt that most readers would be able to relate to at least one person's experience: ‘… I think it's a really good idea to have people talking about their experiences because I think, well like I related to one of them, I think most people will relate to one or more of the things that these people are saying’ (F8, 55y).

However, some participants felt the narratives were not widely generalizable: ‘I'm not disputing that happened to them, but their experience, we're all individuals and we all don't have the same reactions to things and somebody's experience may be very, very different to mine…I would use it as a guide but I wouldn't use it as a ‘well this is what happened to them and that's what's going to happen to me’.’ (M8, 45y).

#### Reassurance

A common theme was that the narratives were reassuring and helped to reduce any fears they had about the test. Some participants commented that they felt ‘relaxed’ and ‘calm’ reading it and it made the test seem a more normal thing to do: ‘My feelings about the experience sheet was one of reassurance, it made me feel like it was a much more normal, not something to be quite so scared about. Not to be anxious about, it's just an everyday thing.’ (F10, 45y); ‘[the narrative information] helps you, and helps people that might be reading it realise that it can be reasonably straightforward and shouldn't necessarily be anything to worry about.’ (M3, 50y). It was also felt that the narrative information could provide reassurance about the perceived unpleasantness of the screening test and reduce the ‘yuck factor’: ‘… it takes some of the fear factor and the yuck factor away in that you think, ‘well these people are just ordinary people and they've been through it’.’ (F8, 55y).

#### Narratives made screening more vivid

Participants describing the narrative information making colorectal screening seem more real was a common theme: ‘…seeing the pictures of the people makes it just a little bit more real I guess.’ (F4, 47y); ‘the testimonials I thought were a really humanising way to let you know: a) you're not alone; and b) it's not as bad as you think. And….. it is worth doing simply because colon cancer is a whole lot worse than……say wiping poo on a card.’ (M8, 45y).

Participants seemed particularly struck by the story of the retired swimming coach who did not initially feel the test was necessary because he had a healthy life style and no colorectal problems, but was found to have cancer: ‘I mean some didn't think they needed it done. He had cancer…. I mean you don't know….. You don't know when you've got it….. I think it's really good, really positive.’ (F1, 52y).

#### Range of experiences presented

Participants reflected that the narrative information presented a good range of responses to the screening invitation: ‘The thing I thought was good was that you have people who had different results and who had different attitudes to the screening, so I thought that was a good spread.’ (F8, 55y); ‘they've all got a different story, how they felt doing it, it's very good.’ (F9, 48y); ‘…..a good cross section of people who were normal, abnormal and actually cancerous…….and particularly in that last article with the lady who refused to have it initially, and then decided later on that she would do. Good.’ (M4, 49y).

### Comparing the factual and narrative information

Both types of information were seen as equally credible, however, one participant did not believe the people in the narrative information were real; ‘when I was reading [it] I did think these are not real people………… I could see myself making up some people because it's much easier than just going out and finding people.’ (M1, 53y). The narrative information was regarded as being more persuasive than the factual information (10/19) although five participants felt the factual information was more persuasive, and responses did not appear to differ by educational achievement or gender. Interestingly, despite the narrative information being viewed as more persuasive by some, this did not seem to be interpreted as being negative or manipulative but more as an acknowledgement that personal stories rather than facts were perceived as more powerful: ‘Perhaps [the narrative information] is more persuasive because it's telling you about people's resistance because they find it unpleasant or a frightening process, and that being set out on paper in front of you kind of challenges you, whereas the facts you can just read it and care to ignore it if you want.’ (M4, 49y).

#### Factual information necessary for making a screening decision but narrative information also important

The factual information being essential to make a decision about screening was a strong theme: ‘I do think that in the interest of people being informed before they give consent to a screening test, you've got to get the facts in.’ (F8, 55y). However, an additional theme that emerged was that participants recognized there were advantages to supplementing the factual information with narrative accounts: ‘…personally for me, the facts were more, sort of resonated a bit more, but I totally understand the benefits of both approaches.’ (M3, 50y); ‘…I definitely don't depend purely on experience in order to go through anything, I want the facts as well, but I think in terms of my health I need both equally.’ (F10, 45y).

#### The narrative information made screening less abstract

A common theme among participants was that having the narrative information made the screening less abstract: ‘…I'd read the facts first because the facts are the facts and you need to know the facts…….. obviously the facts can be dry and a bit abstract whereas the experience gives you that human angle’ (M4, 49y); ‘…you can empathise with the people, there's a danger that if you just have the facts, you can't connect with them…you're looking for a way out basically [of doing the screening]…but if you've got the human experiences, it's kind of these people are just like you, they're no different from you.’ (F6, 47y);

### Improvements to colorectal cancer screening information

The most frequent suggestion (10/19 – 6 women, 4 men) as to how the information could be improved was to provide both types of information to people invited to colorectal cancer screening: ‘…I think a combination of the two is the only thing [that could be done to improve the information], ‘cause…. it resonates in different ways with different people, and I think it would reach a far, far greater number of people with the two of them combined’ (M3, 50y); ‘I think it's foolish not to gather as much information as you can if you're going to make an important decision…..not that I particularly wanted or liked [the narrative] one, but it's again, it's something that allows you, or can contribute to the decision you make, especially if you're struggling with it.’ (M6, 49y).

## Discussion

The use of narrative information in medical decision‐making has been regarded as controversial because of the potential for this type of information to be more powerful or persuasive than traditional factual information.^2^, ^18^ The present study sought to gain a perspective on the use of narrative information from future users of colorectal screening information. People's responses to factual and narrative information about colorectal screening suggested that the factual information was regarded as essential to making a screening decision. However, the addition of narrative information was viewed favourably and recognized as providing a different source of information that could potentially assist the decision‐making process.

The participants tended to make fewer comments about the factual than the narrative information which previous work has also noted.^19^ Narrative information tends to be regarded as more novel than factual information^14^ which may explain why participants felt there was more to comment on. In addition, it was widely acknowledged that the facts provided essential information and so perhaps participants felt there was less need to comment on them. Among the comments on the narratives several key themes emerged: identification, reassurance and vividness which have all been previously acknowledged as functions of narrative information^11^, ^17^, ^25^ and suggests the narratives had the intended impact on the participants.

In terms of participants’ desire to have the factual information supplemented with the narratives, it was clear that the majority of respondents recognised the benefits of both types of information and would ideally like information on colorectal screening to include both factual and narrative information. It is interesting to note that while some of the participants reported that the narrative information was more persuasive this was not interpreted in a negative way and was more an acknowledgement that stories can be more powerful than facts. Given that the factual information was regarded as essential, it seems in this sample that respondents would be unlikely to be persuaded by the narrative information alone and instead regard it as a useful additional information source. This finding is supported by the results of the review of 17 studies by Winterbottom *et al*.^18^ which included first and third person narratives. The review reported that narrative information influenced decision‐making more when compared with no additional information or statistical information in three of seven studies. Although the authors state that the use of narratives to facilitate medical decision‐making should be used with caution, this suggests that narrative information may not necessarily have the detrimental, persuasive impact on decision‐making that some authors have feared.^2^, ^18^

Furthermore, narrative information may be a promising way to communicate information to individuals who may have lower levels of literacy, limited numeracy skills or lower self‐efficacy for understanding health information^11.^ The increasing use of digital information sources in health care, which allows multiple formats of information (e.g. videos, narratives, statistics), may help to reach individuals not engaged by traditional, fact based, health information materials.

The study had limitations. Both the factual and narrative information provided to participants were abridged versions of their original forms. This was done to reduce the burden on participants who were also required to engage in a lengthy discussion after reading the information. It remains possible that if participants had read the full NHS Bowel Cancer Screening Programme leaflet^21^ and had access to all accounts available on www.healthtalk.org their responses may have been different. The narrative information provided only four people's perspectives on colorectal screening and so cannot be regarded as representative. It has previously been acknowledged that experiential information does not accurately reflect the range of experiences in a population.^19^ Additionally, as the focus of the study was to examine ways to potentially increase uptake of screening rather than informed decision‐making, the narratives included were all from individuals who did the screening. However, care was taken in selecting the stories from www.healthtalk.org to try to represent a range of views and feelings about screening. Indeed, participants commented that a good cross section of responses to a screening invitation was presented. Future research may consider presenting different narratives, including a story from a person who declined the offer of screening and a person who had a negative experience of screening to assess people's responses to this. However, it is interesting to note that no participant suggested that these types of experiences should be included.

Another limitation of the study was that we sought the perspective on the use of narrative information from future users of colorectal screening information and did not ask participants about a real decision‐making situation. However, Wyke *et al*.^26^ explored the kinds of information that people need, prefer and use in relation to choice for real health issues (antenatal screening, ending a pregnancy for foetal abnormality, screening for sickle cell disorder or thalassaemia, caring for a person with dementia and lymphoma) and reported similar findings to the present study. This present study also adds a perspective on people's responses to narrative bowel cancer screening information. The majority of participants in this study were of ‘White’ ethnic backgrounds and this may be viewed as a limitation. Previous research has found that ethnic groups, in particular African Americans, benefit more from narrative information than Caucasians,^27^ possibly because African Americans maintain strong storytelling traditions.^28^ Future research could establish if there are ethnic differences in response to factual and narrative information among ethnic minorities in the UK. The majority of participants also had a university‐level education, nonetheless 9/19 had school‐level or no formal qualifications, and responses did not appear to vary considerably by educational attainment.

## Conclusion

Supplementing factual information with patient narratives was positively received by the participants in this study and suggests there may be a use for narrative information when people are considering a screening offer. Currently, the only information people invited to participate in bowel screening receive is factual information. This initial study suggests that some people may find reading about other people's experiences about cancer screening useful. If further work supported our initial findings, the NHS Cancer Screening Programmes could consider supplementing existing fact‐based information with patient narratives when inviting people to participate in cancer screening and possibly sign posting people to resources such as www.healthtalk.org. Narrative information may be particularly important in the context of colorectal screening which is a relatively new addition to the NHS National Screening Programmes, and by its nature people may be less inclined to talk about the process with family and friends. In such cases providing patient narratives may provide emotional and social information which is not typically addressed in routine, factual health information.

How narrative information could be incorporated into the existing UK colorectal cancer screening programmes requires further consideration and research. It may be that the two types of information could be merged into one leaflet with the factual information supplemented by short narrative ‘case studies’ although this would increase the length of the leaflet and could distract readers from the essential factual information. Alternatively, the two types of information could remain as separate leaflets. This has the benefit that participants could then select which information they were most interested in reading but may overload participants with too many pieces of paper and information. The optimal timing of when supplementary narrative information could be given to participants should also be considered in terms of whether it should be included with the initial invitation or when people receive the home‐completed test kit.

## Conflict of interests

None declared.
